# Transcriptome-wide identification and expression analysis of the KT/HAK/KUP family in *Salicornia europaea* L. under varied NaCl and KCl treatments

**DOI:** 10.7717/peerj.12989

**Published:** 2022-03-03

**Authors:** Jia Wei, Richard John Tiika, Guangxin Cui, Yanjun Ma, Hongshan Yang, Huirong Duan

**Affiliations:** 1 Lanzhou Institute of Husbandry and Pharmaceutical Science, Chinese Academy of Agricultural Sciences, Lanzhou, Gansu Province, China; 2 College of Forestry, Gansu Agricultural University, Lanzhou, Gansu Province, China

**Keywords:** *Salicornia europaea*, PacBio Iso-Seq, Halophyte, Plant growth and development, HAK/KUP/KT, MEME analysis, Phylogenetic tree, Gene expression, K^+^ deficiency, Salt treatments

## Abstract

**Background:**

The KT/HAK/KUP (KUP) transporters play important roles in potassium (K^+^) uptake and translocation, regulation of osmotic potential, salt tolerance, root morphogenesis and plant development. However, the KUP family has not been systematically studied in the typical halophyte *Salicornia europaea* L., and the specific expression patterns of *SeKUPs* under NaCl condition and K^+^ deficiency are unknown.

**Methods:**

In this study, *SeKUPs* were screened from PacBio transcriptome data of *Salicornia europaea* L. using bioinformatics. The identification, phylogenetic analysis and prediction of conserved motifs of SeKUPs were extensively explored. Moreover, the expression levels of 24 selected *SeKUPs* were assayed by real-time quantitative polymerase chain reaction (RT-qPCR).

**Results:**

In this study, a total of 24 putative *SeKUPs* were identified in *S. europaea*. Nineteen SeKUPs with the fixed domain EA[ML]FADL were used to construct the phylogenetic tree, and they were divided into four clusters (clusters I–IV). MEME analysis identified 10 motifs in *S. europaea*, and the motif analysis suggested that 19 of the identified SeKUPs had at least four K^+^ transporter motifs existed in all SeKUPs (with the exception of SeKUP-2). The RT-qPCR analysis showed that the expression levels of most *SeKUPs* were significantly up-regulated in *S. europaea* when they were exposed to K^+^ deficiency and high salinity, implying that these SeKUPs may play a key role in the absorption and transport of K^+^ and Na^+^ in *S. europaea*.

**Discussions:**

Our results laid the foundation for revealing the salt tolerance mechanism of SeKUPs, and provided key candidate genes for further studies on the function of KUP family in *S. europaea*.

## Introduction

Salt stress is one of the most important environmental factors affecting plant growth and development (Nabati et al., 2011). The excessive salt concentration in soil causes reduction in water potential, ions toxicity, osmotic stresses and induced secondary stress which even lead to plants’ death (Munns & Tester, 2008). Halophytes are a special plant species: they can complete their life cycle in a saline environment of at least 200 mM NaCl condition (Flowers, Glenn & Volkov, 2019). Most halophytes are able to maintain the relative stability of potassium ion (K^+^) content in the above-ground organs of plants in a high salt concentration environment (Flowers, Troke & Yeo, 1977), such as *Lycium ruthenicum* (Dai et al., 2019), *Phragmites australis* (Takahashi et al., 2007a) and *Mesembryanthemum crystallinum* (Su et al., 2002).

K^+^ is an essential mineral for plant growth and development and is also the most abundant monovalent cation in plants, accounting for approximately 2% to 10% of plant dry weight (Clarkson & Hanson, 1980), and it plays a significant role in various physiological and biochemical processes, for instance, abiotic stress adaptation, stomatal movement, enzyme function and signal transduction (Véry & Sentenac, 2003). According to the transport characteristics of K^+^, K^+^transport families are divided into four types: Trk/HKT (tandem-pore K^+^ channels) family, KT (K^+^transporter)/HAK (high-affinity K^+^)/KUP (K^+^uptake) family, CHX (cation/hydrogen exchanger) family and KEA (K^+^ efflux anti-porter) family (Gupta et al., 2008; Mäser et al., 2001). Among them, the KT/HAK/KUP (KUP) family belonging to the APC (amino acid polyamine organization) superfamily, is the largest and widely distributed in bacteria, fungi, and plants, but has not yet been identified in animal cells (Corratgé-Faillie et al., 2010). The KUP transporters were first identified in *Arabidopsis thaliana* (*KUP1/KT1* and *KUP2/KT2*) and *Hordeum vulgare* (*HAK1*); thus, the composite name KUP, is widely used to refer to the whole family in plants (Véry et al., 2014; Epstein & Kim, 1971; Bañuelos et al., 1995). In the early stage, the KUP family was divided into four clusters (I–IV) (Rubio, Guillermo & Alonso, 2010). Recently, researchers discovered that this family has been re-divided into five clusters (clusters I–V) (Nieves-Cordones et al., 2016), and the main reason for this phenomenon is due to species diversity.

Firstly, the different cluster members have different physiological functions. The cluster I members can improve the absorption capacity of root system to K^+^ under K^+^ deficiency condition, such as AtHAK5 (*A. thaliana*), OsHAK1 (*Oryza sativa*) and SiHAK1 (*Setaria italica*) (Rubio, Guillermo & Alonso, 2010; Zhang et al., 2018). Members of cluster II have diverse functions in plant growth and development. For example, VvKUP2 (*Vitis vinifera*) can promote the expansion of berry epidermal cells (Davies et al., 2006; Elumalai, Nagpal & Reed, 2002). The members of cluster III can maintain K^+^/Na^+^ homeostasis, like HcKUP12 (*Halostachys capsica*) (Yang & Wang, 2015) and PhaHAK5 (*Phragmites australis*) (Takahashi et al., 2007b). However, members in clusters IV and V have not yet been adequately studied (Bañuelos et al., 2002). Furthermore, some KUPs have been demonstrated to protect plants against salt stress. For instance, the constitutive overexpression of OsHAK5 in tobacco improved K^+^ accumulation under salt stress (Elumalai, Nagpal & Reed, 2002). AtHAK11 and McHAK2 (*Mesembryanthemum crystallinum*) can promote the uptake of K^+^ when plants are under salt stress (Su et al., 2002; Maathuis, 2006). These data indicate that members of the KUP family play critical roles in the uptake and transport of K^+^ and in regulation of plant growth, development, and abiotic stress tolerance.

*Salicornia europaea* L., a succulent halophyte, belongs to the family of *Amaranthaceae*, and it is a typical salt-resistant predominant species in the world (Nikalje et al., 2018). In the long-term evolutionary process, this special plant has gradually formed a strong salt tolerance mechanism in extremely saline environments. It can tolerate soil with more than 1,000 mM NaCl (Flowers & Colmer, 2008; Ozawa, Jianmei & Fujii, 2007), also accumulates large amounts of Na^+^ than K^+^ and compartmentalize Na^+^ in the vacuole (Lv et al., 2012). Meanwhile, some research results show that *S. europaea* can still maintain a relatively stable K^+^/Na^+^even under increasing salt concentrations and longer treatment time (Wang et al., 2009; Fan et al., 2013), implying that *S. europaea* has a strong K^+^ transport system under salt condition. Therefore, it is meaningful to elucidate the mechanism of K^+^ uptake in *S. europaea*. However, the information about K^+^ uptake family in *S. europaea* remains unknown.

In this study, we identified *SeKUPs* in *S. europaea* using PacBio sequencing system data (Tiika et al., 2021). We thoroughly performed multiple sequence alignment, presence of conserved motifs in the proteins, phylogenetic analysis, and real-time quantitative polymerase chain reaction (RT-qPCR) of *SeKUPs* in different tissues of *S. europaea* in response to salinity and K^+^ deficiency. This study provides an important theoretical basis for the mechanism of K ^+^ uptake in *S. europaea*.

## Materials & Methods

### Plant materials and treatments

The wild seeds of *S. europaea* were collected from Liangcao Village, Jingtai County, Baiyin City, Gansu Province in China (37°21′2″N, 104°5′28″W). The seeds were disinfected with 2% NaClO solution for about 3 min and washed with distilled water and then germinated at 28 °C on filter paper in the dark for 72 h. The plantlets were transferred into containers with sterilized sand, and were irrigated with 1/2 Hoagland nutrient solution (pH = 5.7). The formulation of 1/2 Hoagland nutrient solution was: 2 mM KNO_3_, 0.5 mM KH_2_PO_4_, 0.5 mM MgSO_4_⋅7H_2_O, 0.5 mM Ca(NO_3_)_2_⋅4H_2_, 50 µM H_3_BO_3_, 10 µM MnCl_2_⋅4H_2_O, 1.6 µMZnSO_4_⋅ 7H_2_O, 0.6 µM CuSO_4_, 0.05 µM Na_2_MoO_4_⋅ 2H_2_O, 0.06 mM Fe-citrate ⋅2H_2_O. All plantlets were grown in an artificial climate box with a temperature of 22 ± 2 °C, relative humidity of about 65% and a daily photoperiod of 16/8 h (day/night; the flux density was approximately 600 µmol/m^2^ s). The nutrient solution was renewed every 3 days.

The four week old plantlets were subjected to NaCl and K^+^ treatments. The seedlings were exposed to 1/2 Hoagland nutrient solution plus NaCl (0 mM, 50 mM and 200 mM) for a period of 0 h, 6 h, 24 h and 48 h, respectively. For K^+^ treatment, the seedlings were exposed to modified 1/2 Hoagland nutrient solution (2 mM KNO_3_ was substituted by 2 mM HNO_3_, 0.5 mM KH_2_PO_4_ was substituted by 0.5 mM H_3_PO_4_) plus 0.01 mM KCl (K^+^ deficiency) or 2.5 mM KCl (normal K ^+^) for 0 h, 6 h, 24 h, and 48 h. Samples of shoots and roots were collected separately and quickly frozen in liquid nitrogen, and stored at −80 °C for RNA extraction.

### Identification of the *SeKUPs* in *S. europaea*

We downloaded the transcriptome sequence of *S. europaea* from the NCBI database (https://www.ncbi.nlm.nih.gov/sra) (Accession number: PRJNA725943) (Tiika et al., 2021). Keywords related to potassium transport proteins were used to search candidate *SeKUPs* in *S. europaea* based on the transcriptome database. The amino acids of SeKUPs were predicted by finder searches for open reading frames (ORFs) (https://www.ncbi.nlm.nih.gov/gorf/gorf.html), then were further identified (*E*-value <1e^−5^) by Blastp (protein-protein BLAST) search from NCBI. Finally, the sequences were subjected to conserved domains validation by InterProScan. We numbered the candidate SeKUPs by using the same overlapping prefix “Se” for *S. europaea*.

### Sequence analyses of *SeKUPs*

The protein sequence of SeKUPs was translated by ORFs (Tian et al., 2019). The biochemical properties of the candidates *SeKUPs* were predicted using the ExPASy (Expert Protein Analysis System) tool (https://web.expasy.org/protparam/) (Feng et al., 2020), including the molecular weight (MW), isoelectric points (pI), extinction coefficients, estimated half-life, instability index, aliphatic index, and grand average of hydropathicity (GRAVY) (Gasteiger et al., 2003).

The conserved motifs of the deduced SeKUPs proteins were identified through MEME (Multiple Expectation Maximization for motif Elicitation) version 5.3.3 ( http://meme-suite.org/doc/cite.html) using the following parameters: the number of motifs searched was set to 10 and the range of the motif length was set to 5–50 aa (Bailey et al., 2015). All motifs were further annotated with InterProScan (http://www.ebi.ac.uk/interpro/) (Mulder & Apweiler, 2007).

### Phylogenetic analysis of *SeKUPs*

The protein sequences for 13 AtKUPs, 27 OsHAKs, 17 ZmHAKs, and 27 VvKUPs were retrieved from NCBI (https://www.ncbi.nlm.nih.govorg), UniPort (https://www.uniprot.org) (Consortium, 2015) and maize website (https://maizesequence.org/), and the newly identified SeKUPs were selected to construct the phylogenetic tree by using MEGA 5.0 with 1,000 bootstrap replications of maximum likelihood. The phylogenetic tree was embellished by the online tool iTOL (http://itol.embl.de/).

### RT-qPCR analysis

Total RNA was extracted from root and shoot tissues using TransZol Up Plus RNA Kit (Lot#M31018) referring to the manufacturer’s instructions. The RNA quantity and quality were determined using a TGen Spectrophotometer (TianGen) based on the A260 nm/A280 nm and A260 nm/A230 nm ratio. *Evo* M-MLV RT Kit (AG11705, Accurate Biotechnology) was used to reverse transcribe the total RNA into cDNA and for removal of genomic DNA mixed in the cDNA before RT-qPCR analysis, following the manufacturer’s protocol.

For RT-qPCR analysis, primers were designed based on mRNA sequences, using Primer 5.0 software and synthesized by TsingKe Biological Technology Co., Ltd. (Xi’an, China). The *S. europaea Ubiquitin-conjugating* (*SeUBC*) gene was used as the reference gene (Xiao et al., 2015). Three biological repeats were conducted and triplicate quantitative assays for each replicate were performed on 0.5 µL of each cDNA dilution using Heiff^®^ qPCR SYBR^®^ Green Master Mix kit (Yeasen Biotech Co., Ltd) per the manufacturer’s protocol. The RT-qPCR analysis was performed using the QuantStudio™ 5 Real-Time PCR Instrument (ABI). Cycling parameters were: 95 °C for 5 min, 40 cycles at 95 °C for 10 s, and 60 °C for 30 s. The relative expression of the *SeKUPs* was calculated according to 2^−ΔΔCt^ (Livak & Schmittgen, 2001). The primer sequences for the *SeKUPs* and the housekeeping gene are listed in Table S1, and some *SeKUPs* share a pair of primers.

### Data analysis

All values reported under gene expression levels are presented as means ± SE (*n* = 3). The significance level among means was analyzed by Duncan’s multiple range tests (*P* ≤ 0.05) after performing a one-way ANOVA analysis using SPSS statistical software (Ver. 25.0, SPSS Inc., Chicago, IL, USA), and all histograms were generated using GraphPad Prism8.0.

## Results

### Identification of *SeKUPs* in *S. europaea*

A total of 24 putative *SeKUPs* were obtained from *S. europaea*, which were designated as *SeKUP1* - *SeKUP24* based on the Blastp results of other plant KUP protein sequences as queries. Among the 24 *SeKUPs*, the predicted cDNA length varied from 1,641 bp (*SeKUP-2*) to 3,391 bp (*SeKUP-24*), and the predicted protein length varied from 101 bp (*SeKUP-2*) to 845 bp (*SeKUP-6, -7, -8,* and *-9*) (Table 1).

**Table 1:** The statistic information of *SeKUPs* in *S. europaea*.

*Gene name*	*Gene ID*	*cDNA length (bp)*	*Span on master (bp)*	*ORF length (bp)*	*Clusters*	*Catalytic Site*	*Full length (“+”)*
*SeKUP1*	*i3_HQ_samplee11669_c30301_f2p0_3024*	*3024*	*2319*	*773*	II	EAMFADL	+
*SeKUP2*	i1_HQ_samplee11669_c10009_f2p0_1641	1,641	471	101	II	EAMFADL	
*SeKUP3*	i2_HQ_samplee11669_c6179_f2p0_2898	2,898	2,367	788	II	EAMFADL	+
*SeKUP4*	i2_HQ_samplee11669_c91677_f3p0_2821	2,821	2,364	787	II	EAMFADL	+
*SeKUP5*	i2_HQ_samplee11669_c107214_f19p1_2853	2,853	2,349	782	II	EAMFADI	
*SeKUP6*	i2_HQ_samplee11669_c2358_f3p1_2778	2,778	2,538	845	I	EAMFADL	+
*SeKUP7*	i3_HQ_samplee11669_c18328_f5p0_3240	3,240	2,538	845	I	EAMFADL	+
*SeKUP8*	i3_HQ_samplee11669_c30205_f2p0_3366	3,366	2,538	845	I	EAMFADL	+
*SeKUP9*	i3_HQ_samplee11669_c4886_f2p0_3368	3,368	2,538	845	I	EAMFADL	+
*SeKUP10*	i2_HQ_samplee11669_c20423_f4p1_2931	2,931	2,322	773	II	EAMFADL	+
*SeKUP11*	i2_HQ_samplee11669_c2189_f7p2_2995	2,995	2,322	773	II	EAMFADL	+
*SeKUP12*	i2_HQ_samplee11669_c23402_f3p1_2760	2,760	2,319	772	IV	EAMFADL	+
*SeKUP13*	i3_HQ_samplee11669_c30270_f2p4_3017	3,017	1,287	428	II	EAMFADL	
*SeKUP14*	i2_HQ_samplee11669_c133022_f3p1_2819	2,819	2,370	789	III	EAMFADL	+
*SeKUP15*	i2_HQ_samplee11669_c132452_f4p1_2805	2,805	2,367	788	III	EAMFADL	+
*SeKUP16*	i3_HQ_samplee11669_c15356_f3p0_3023	3,023	1,845	614	III	EAMFADL	
*SeKUP17*	i2_HQ_samplee11669_c131282_f2p0_2417	2,417	2,181	726	II	EAMFADL	
*SeKUP18*	i2_HQ_samplee11669_c190749_f5p0_2855	2,855	2,181	726	II	EAMFADL	
*SeKUP19*	i2_HQ_samplee11669_c193357_f2p0_2845	2,845	2,181	726	II	EAMFADL	
*SeKUP20*	i1_HQ_samplee11669_c182924_f2p0_1885	1,885	1,857	618	–	KNDNITK	
*SeKUP21*	i1_HQ_samplee11669_c11500_f7p1_1991	1,991	1,251	416	–	AILLGIT	
*SeKUP22*	i1_HQ_samplee11669_c127136_f2p1_1797	1,797	1,251	416	–	AILLGIT	
*SeKUP23*	i3_HQ_samplee11669_c18353_f6p0_3074	3,074	2,148	715	–	EDFDTEE	
*SeKUP24*	i3_HQ_samplee11669_c19195_f2p0_3391	3,391	1,008	335	–	–	

To further analyze the characteristics of SeKUPs with complete sequences, a total of 12 SeKUPs were predicted by ORF finder, including SeKUP-1, -3, -4, -6, -7, -8, -9, -10, -11, -12, -14 and -15 (Table S2). Then the MWs, pIs, estimated half-life, instability index, aliphatic index, and GRAVY of these 12 SeKUPs were also calculated. As shown in Table S2, the ORF protein length of the predicted 12 SeKUPs ranged from 772 bp (SeKUP-12) to 845 bp (SeKUP-6, -7, -8 and -9); the molecular weight (MW) varied from 86,940.07 kDa (SeKUP-1, -10, and -12) to 94,683.36 kDa (SeKUP-8); isoelectric point (pI) varied from 5.79 (SeKUP-6, -7, -9) to 8.08 (SeKUP-1, -10, -11, and -12), extinction coefficients varied from 107,565 (SeKUP-8) to 128,660 (SeKUP-3, -4), all estimated half-life >10 h, the instability index of 12 SeKUPs proteins was lower than 42 (with the exception of SeKUP-3), the aliphatic index varied from 105.5 (SeKUP-8) to 111.64 (SeKUP-15), and the GRAVY varied from 0.222 (SeKUP-11) to 0.367 (SeKUP-14, -15), respectively (Table S2). In summary, most SeKUPs of the same subfamily shared similar sequences characteristics (MW, pI estimated half-life, instability index, aliphatic index, and GRAVY).

### Phylogenetic analysis of SeKUPs

**Figure 1:**
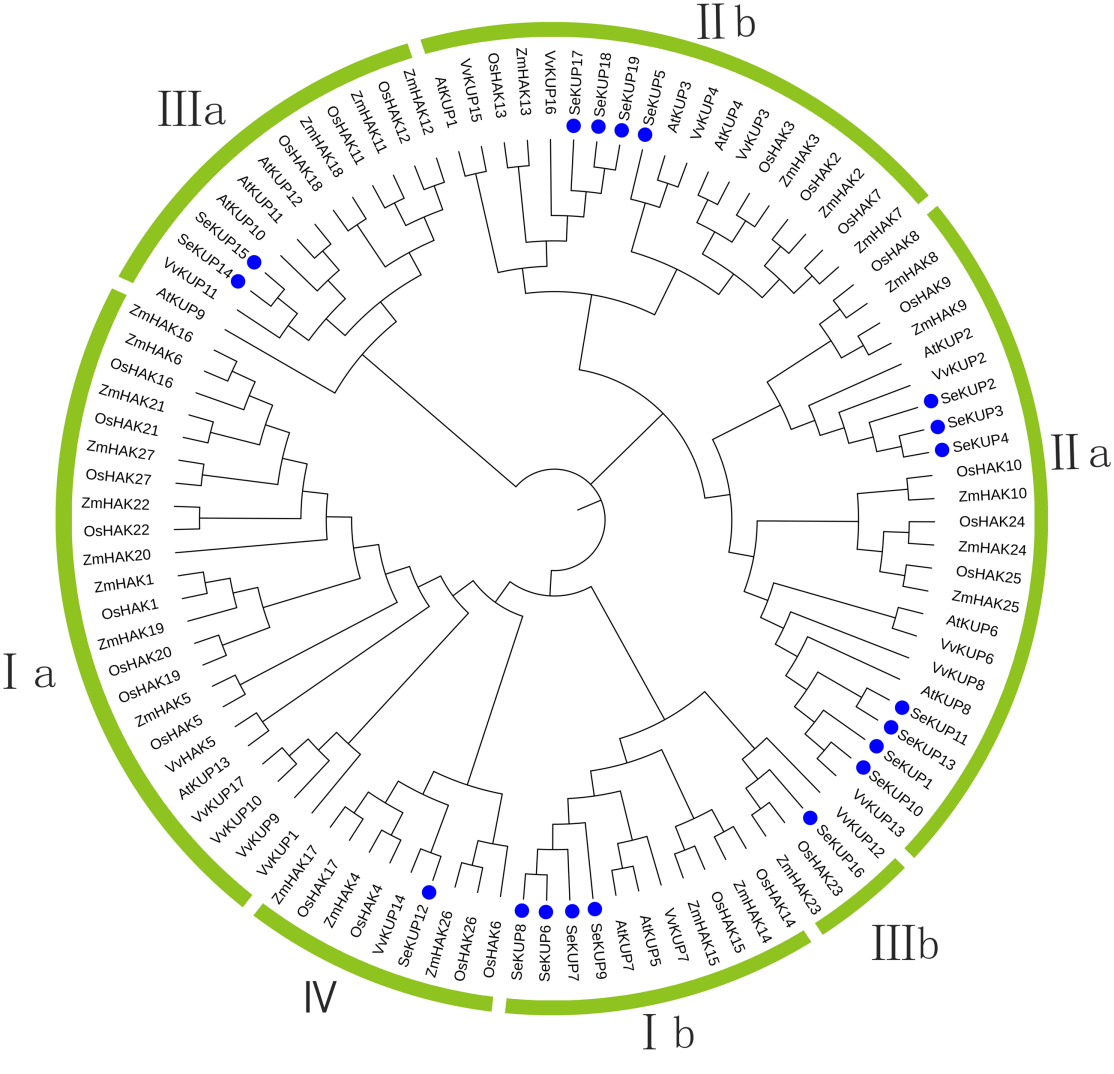
Phylogenetic tree of KUP family from *S. europaea*, *A. thaliana*, *O. sativa*, *V. vinifera* and *Z. mays*. Nineteen (19) SeKUPs are marked with blue circles. Groups I, II, III, IV represent the four clusters, a and b represent corresponding subgroups.

For the KUP family, it is generally believed that the sequence containing the EA [ML] FADL motif is identified as a KUP member (Ou et al., 2018). Therefore, through analysis, 19 sequences in our data contain this motf. So we use these 19 sequences to construct the evolutionary tree. These SeKUPs were divided into four clusters (cluster I, II, III, and IV) by phylogenetic analysis (Fig. 1) through other KUP protein sequences from four model plants (Table S3). In addition, the SeKUPs members in clusters I, II, and III were further classified into sub-clusters Ia, Ib, IIa, IIb, and IIIa, IIIb, respectively. SeKUP -6, -7, -8, and -9 belonged to cluster I; SeKUP -1, -2, -3, -4, -5, -10, -11, -13, -17, -18, and -19 belonged to cluster II; SeKUP -14, -15, -16 belonged to cluster III and SeKUP-12 belonged to cluster IV (Fig. 1). In summary, cluster II is the most abundant and cluster IV is the least abundant in *S. europaea*.

### Conserved motif analysis of SeKUPs

**Figure 2:**
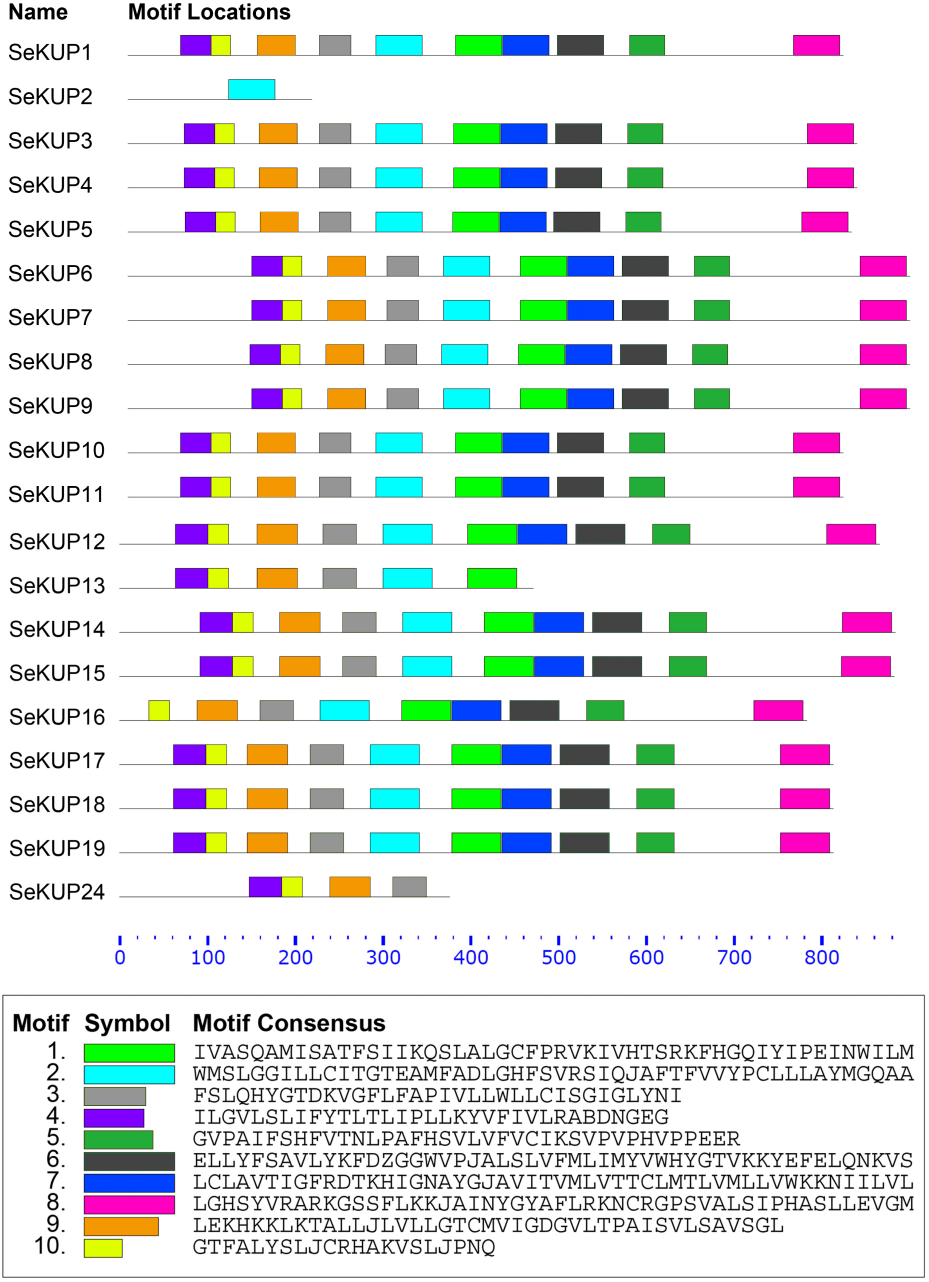
Motif analysis of SeKUPs in *S. europaea*. Conserved motifs of the SeKUPs protein are investigated on the MEME web server, and are named as motif 1 to 10 with different colors. The different colored boxes represent different motifs and their position in each SeKUP sequence. Each motif is indicated by a colored box in the legend at the bottom. The parameter 0–800 is the sequence length of amino acids. New figure legends have been submitted in the system.

A total of 10 conserved motifs in putative SeKUP proteins were identified and designated as motifs (1–10) (Fig. 2). More detailed information on all conserved motifs can be found in Table S4. As revealed by our InterProScan search, most of the conserved motifs were found within the sequence of the K^+^ transporters, with the exception of motif 10 (Table S4). As shown in Fig. 2, with the exception of SeKUP-2, all the identified SeKUPs contained at least four K^+^ transporter motifs. In addition, motifs 1, 2, 6, 7, and 8 had the most amino acid sequences, whereas motif 10 contained the fewest protein sequences (Table S4). The majority of SeKUPs proteins contain the same types of motifs.

### Expression patterns of *SeKUPs* under K^+^ deficiency

Previous studies have demonstrated that the expression levels of KUPs are generally regulated by the concentration of K^+^ (Zhou et al., 2020), such as OsHAK1 (Chen et al., 2015) in *O. sativa* and TaHAK1 in *Triticum aestivum* (Cheng et al., 2018). In addition, further studies have shown that members of the KUP family play an important role in the high affinity K^+^ uptake process under K^+^ deficiency condition (Rubio, Guillermo & Alonso, 2010; Santa-María, Oliferuk & Moriconi, 2018). To investigate the expression divergence of *SeKUP* s in response to different K^+^ conditions in *S. europaea*, we analyzed the expression patterns of *SeKUPs* in the roots and in the shoots under 2.5 mM KCl (normal growth K^+^ condition) and 0.01 mM KCl (K^+^ deficiency) for different time periods (Table S5).

**Figure 3:**
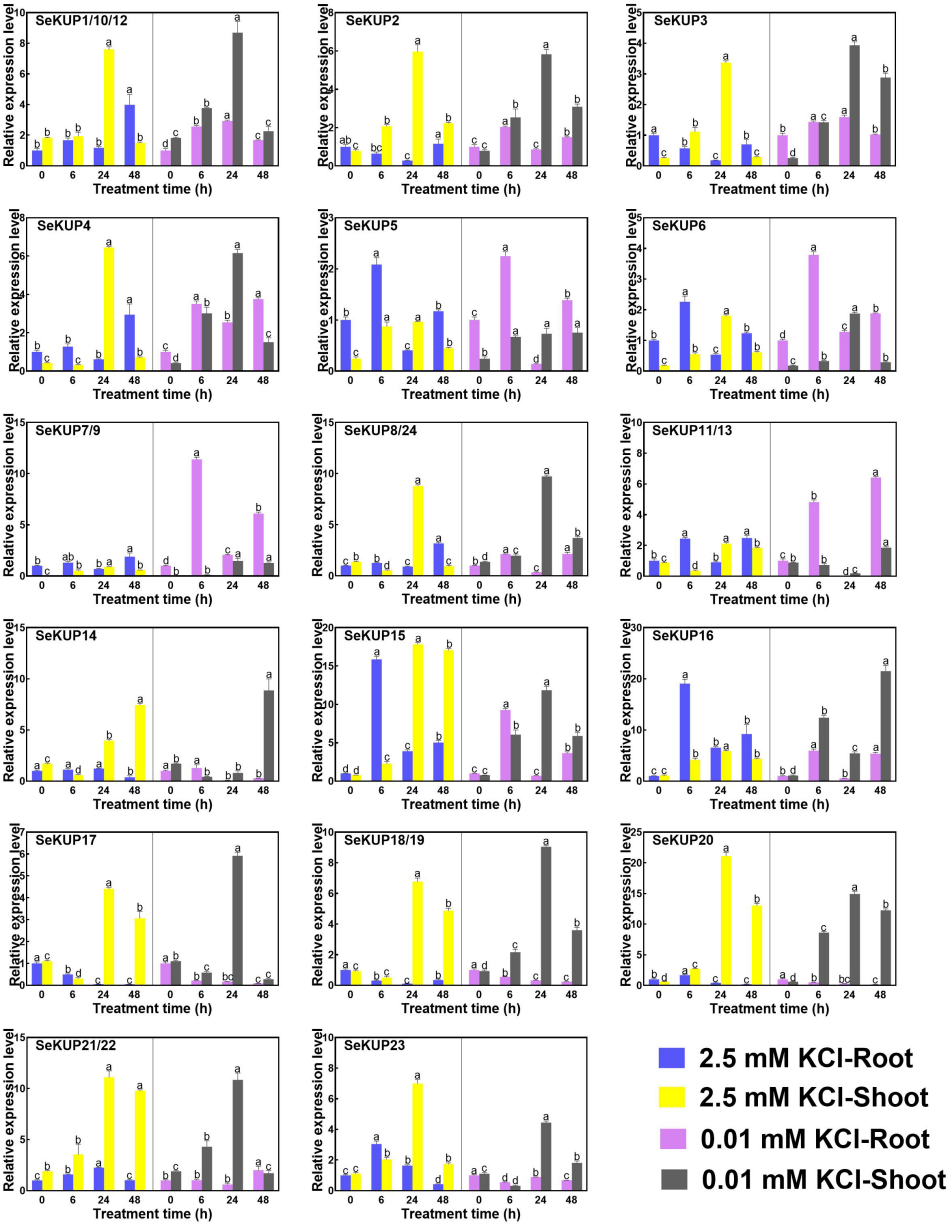
Real-time quantitative polymerase chain reaction (RT-qPCR) analysis of *SeKUPs* in *S. europaea* under KCl treatments. Values are means ± standard errors (SEs) (*n* = 3) and bars indicate SEs. Different letters (Duncan’s test, *p* < 0.05) reflect the significant differences among different treatment times under the same KCl concentration, respectively. The gene name is on the top left of each column graph. The seedlings of *S. europaea* are grown in the 1/2 Hoagland nutrient solution, and four weeks old seedlings are treated with modified 1/2 Hoagland nutrient solution plus different KCl concentrations for varied times. In the modified 1/2 Hoagland nutrient solution, 2 mM KNO_3_ is substituted by 2 mM HNO_3_, 0.5 mM KH_2_PO_4_ is substituted by 0.5 mM H_3_PO_4_. The relative expression levels of all *SeKUPs* are calculated by 2^−ΔΔCt^ method, and 2.5 mM KCl-0 h-root is used as the standard control. Some *SeKUPs* with “/” represent that they shared the same primers and the same expression patterns.

Under the normal growth condition (2.5 mM KCl), the majority of *SeKUPs* were expressed in both shoots and roots, except for *SeKUP-7/9*, which was mainly expressed in the shoots. With the extension of treatment time under 2.5 mM KCl, most of the *SeKUPs* were induced more in the shoots than in the roots, and peaked at 24 h of treatment (Fig. 3). Under K^+^ deficiency condition, some *SeKUPs* were induced significantly compared to the control (2.5 mM KCl treatment) both in the roots and shoots with time prolonged, for example, SeKUP*-1/10/12, -2, -3, -4, -7/9, -8/24, -17* and *-18/19*, and the relative expression levels of *SeKUP-3* in the roots (R)-24 h, *SeKUP-3* in the shoots (S)-48 h, *SeKUP-4* in S-6 h and *SeKUP-7/9* in R-6 h were 8.7, 9.9, 9.6 and 8.7 times higher than their respective controls. Differently, *SeKUP-6* showed induced expression in the roots and inhibited expression in the shoots. Besides, some *SeKUPs* like *SeKUP-15*, *-16*, *-20*, *-21/22* exhibited reduced expression in the roots, but they were induced significantly in the shoots than control at short time treatment (6 h). Compared to the control, the expression of *SeKUP-23* under K^+^ deficiency were reduced at 6 h and 24 h treatment, then returned to the normal expression at 48 h treatment.

### Expression patterns of *SeKUPs* under NaCl treatments

**Figure 4:**
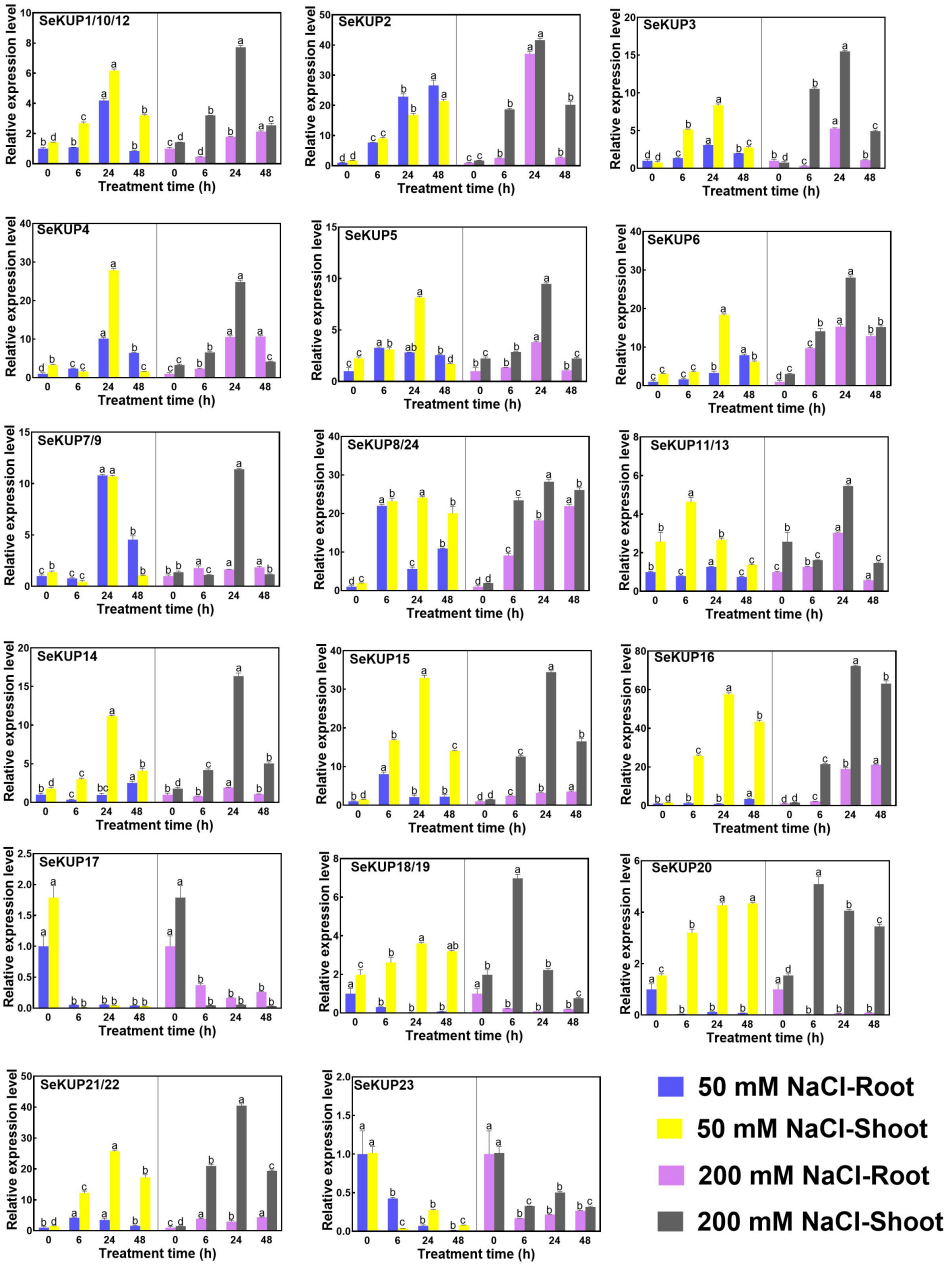
Real-time quantitative polymerase chain reaction (RT-qPCR) analysis of *SeKUPs* in *S. europaea* under under NaCl treatments. Values are means ± standard errors (SEs) (*n* = 3) and bars indicate SEs. Different letters (Duncan’s test, *p* < 0.05) reflect the significant differences among different treatment times under the same NaCl concentration, respectively. The gene name is on the top left of each column graph. The seedlings of *S. europaea* are grown in the 1/2 Hoagland nutrient solution, and four weeks old seedlings are treated with 1/2 Hoagland nutrient solution plus different NaCl concentrations for varied times. The relative expression levels of all *SeKUPs* are calculated by 2^−ΔΔCt^ method, and 0 mM NaCl-0 h-root is used as the standard control. Some *SeKUPs* with “/” represent that they shared the same primers and the same expression patterns.

Studies have found that members of the KUP family are also involved in plant salt stress responses and regulate salt tolerance through a series of mechanisms (Chen et al., 2015; Horie et al., 2011). To further analyze the expression patterns of *SeKUPs* under salinity, we exposed the seedlings to 50 mM NaCl and 200 mM NaCl treatments for different times (0 h, 6 h, 24 h and 48 h) (Table S6, Fig. 4).

With NaCl application, the majority of *SeKUPs* were induced in both shoots and roots, and the relative expression levels increased with time, and then peaked at 24 h, except for *SeKUP-17* and *23* (expression was inhibited in both shoots and roots). Notably, the transcript abundance of *SeKUP-2, -3, -6, -8/24, -15, -16* was 6.1 to 36.5 fold higher in the shoots under 50 mM NaCl condition at 24 h than at 0 h, and the values under 200 mM NaCl condition at 24 h were 9.3 to 45.7 fold higher compared to 0 h. Some *SeKUPs* like *SeKUP-18/19* and *SeKUP-20* were inhibited in the roots, while they were induced significantly in the shoots with increasing time under 50 mM and 200 mM NaCl treatments. Compared with 50 mM NaCl treatment, 200 mM NaCl significantly induced the expression of *SeKUP-2, -3, -6, -14, -18/19,* and *-21/22* in the shoots.

## Discussion

Given their key roles in plant K^+^ uptake, homeostasis, translocation, stress resistance, and development, the KUP family has been identified in many plant species such as *A. thaliana* (Ahn, Shin & Schachtman, 2004), *O. sativa* (Gupta et al., 2008), *Zea mays* (Zhang et al., 2012) and *Pyrus bretschneideri* (Yan et al., 2018) genomes, respectively. In our study, we identified 24 *SeKUPs* from *S. europaea* (Table 1), compared with *A. thaliana* (13) (Ahn, Shin & Schachtman, 2004), *V. vinifera* (17) (Davies et al., 2006), *Prunus persica* (16) (Song, Ma & Yu, 2015), and *Solanum lycopersicum* (19) (Hyun et al., 2014), the number of *SeKUPs* is similar to or more than other plants, providing useful information for further functional validation of SeKUPs in *S. europaea*.

Conserved domains are the core of a protein family and have important functions in genes. At present, several typical conserved protein domains have been found in KUP family members, such as GVVYGDLGTSPLY (Rodríguez-Navarro, 2000) and LAYMGQAA, but the conserved domains vary among species. Although the conserved structure of KUP family is different, it has some relatively conserved amino acid domains, the highly conserved domains were searched by motif analysis to speculate whether these family members have functional differences during evolution (Wang et al., 2018). The results showed that 19 SeKUPs had the same conserved domain EA [ML] FADL, which means these sequences are highly conserved and facilitate phylogenetic tree analysis. In our study, we found 19 SeKUPs with the fixed domain EA[ML]FADL, this domain also appeared in *Manihot esculenta* (Ou et al., 2018), *P. bretschneideri* (Wang et al., 2018) *P. persica* (Song, Ma & Yu, 2015) and *T. aestivum* (Cheng et al., 2018)*.* MEME revealed motifs that are conserved in the proteins originating from all four clusters. We searched for 10 motifs in *S. europaea* and 90% belonged to K^+^ transporters. Motif analysis suggested that 19 of the identified SeKUPs had at least four typical motifs of K^+^ transporters, with the exception of SeKUP2. A similar phenomenon also appeared in the motif analysis of *O. sativa* (Gupta et al., 2008). Although some homologous SeKUPs had different motifs structures (such as SeKUP-2/13), the majority of SeKUPs within the same subgroup shared similar motifs, and a similar number of motifs were present in SeKUPs proteins from each of the four clusters, indicating that the classification of SeKUPs was further supported by conserved motifs, with each subgroup sharing similar motifs. In *S. europaea*, with the exception of SeKUP-2, -13, -16, and -24, all SeKUPs contained 10 motifs, this phenomenon is similar to results in *M. esculenta*, where all MeKUPs contained 16 motifs with the exception of MeKUP-1, -7, -9, -10, -13, -15, -16, and -17 (Ou et al., 2018). These results support the high conservatism of sequences among KUP subgroup members.

In our study, 19 SeKUPs were classified into four clusters based on their evolutionary relationships (Fig. 2). This is consistent with previous classifications of the KUP family in *A. thaliana*, *O. sativa*, *V. vinifera* and *Z. mays* (Zhang et al., 2012; Gupta et al., 2008). The results showed that most of the *SeKUPs* members were concentrated in cluster II. The number distribution of KUPs in the four clusters varied greatly, but this situation was consistent with previous studies that distributed KUPs unevenly in different clusters among angiosperms (Nieves-Cordones et al., 2016). Previous studies indicated that *KUPs* are widely expressed in different tissues of plants, such as roots, stems, leaves, flowers, and fruits (Corratgé-Faillie et al., 2010; Ahn, Shin & Schachtman, 2004). In the present study, we observed the consistent phenomenon that most *SeKUPs* were expressed in the shoots and roots, implying that they might play important roles in both shoots and roots. Besides, *SeKUPs* in the same cluster exhibited similar expression patterns. The representative members of cluster I, such as OsHAK1*,* OsHAK5 in *O. sativa* (Chen et al., 2015), and PbrHAK1 in *P. bretschneideri* (Wang et al., 2018) were reported to be induced by K^+^starvation, and they mainly mediate high affinity K^+^ transport. SeKUP-6, -7*,* and -9 belonged to cluster I, and their expression abundance was significantly induced under K^+^ deficiency than under normal K^+^ condition, especially in the roots, implying that they might be mainly responsible for K^+^ transport with high affinity. Meanwhile, the expression patterns of *SeKUPs* members from cluster II showed similar changes under normal K^+^ condition and under K^+^ deficiency (Gupta et al., 2008; Rubio, Guillermo & Alonso, 2010; Santa-María, Oliferuk & Moriconi, 2018). Previous reports have shown that members of the cluster II, for example, HvHAK2 in barley, AtKUP1, and AtKUP2 have different K^+^ transport activities in dicotyledons (Véry et al., 2014). Our consistent results revealed that SeKUPs members from cluster II might be simultaneously involved in high-affinity and low-affinity K^+^ absorption, and our speculation needs to be further validated. Transcriptional regulation of K^+^ transporter genes represents a major mechanism in plant responses to K^+^ deficiency, and expression pattern analysis can provide insight into the potential functions of the SeKUPs in *S. europaea*.

The expression of *SeKUPs* was affected not only by the concentration of K^+^ in the medium, but also by NaCl in the medium. Similar phenomenon also occurred in other plants with KUPs (Ou et al., 2018; Cheng et al., 2018), implying that these up-regulated KUPs may play a potential role under salt stress. In our study, we found that 22 *SeKUPs* were significantly up-regulated by salt stress, indicating that they could play a potential function under NaCl treatment, and further functional verification need to be explored among them. The up-regulation of *SeKUP-16* was the most significant compared with the control, suggesting that *SeKUP-16* might be a candidate gene for the adaptation of *S. europaea* to saline environment (Fig. 4). In addition, we found that the expression levels of some *SeKUPs* differed between shoots and roots, and that some *SeKUPs* were greatly suppressed under salt treatments. This is similar to *Camellia sinensis* (*CsHAK17*) (Yang et al., 2020), implying that they are not sensitive to high NaCl and K^+^ deficiency.

## Conclusions

In this study, we use Pac-Bio sequencing transcriptome data to discover 24 *SeKUPs* from *S. europaea*. Through conservative domain verification and motif analysis, we found that 19 SeKUPs have a fixed domain sequence (EA[ML]FADL) and were used to construct phylogenetic tree. Based on the phylogenetic relationships, the 19 SeKUPs could be divided into four clusters: I, II, III, and IV, in addition, clusters I to III were subdivided into subclusters a and b, rspectively. The RT-qPCR further validated the key role of 24 *SeKUPs* under abiotic stresses (salt and K^+^ deficiency) in *S. europaea*.

## Supplemental Information

10.7717/peerj.12989/supp-1 Supplemental Information 1 Primers used in Real-time quantitative polymerase chain reaction (RT-qPCR) analysis

10.7717/peerj.12989/supp-2 Supplemental Information 2 Characteristics of full-length sequence SeKUPs

10.7717/peerj.12989/supp-3 Supplemental Information 3 The accession number of KUP genes in *Arabidopsis thaliana*, *Oryza sativa*, *Vitis vinifera* and *Zea mays*

10.7717/peerj.12989/supp-4 Supplemental Information 4 Conserved amino acid motifs and annotation of SeKUPs

10.7717/peerj.12989/supp-5 Supplemental Information 5 Real-time quantitative polymerase chain reaction (RT-qPCR) analysis of *SeKUPs* in *S. europaea* under KCl treatments

10.7717/peerj.12989/supp-6 Supplemental Information 6 Real-time quantitative polymerase chain reaction (RT-qPCR) analysis of *SeKUPs* in *S. europaea* under NaCl treatments

## Additional Information and Declarations

### Data Availability

The following information was supplied regarding data availability: The raw data is available in the Supplementary Files.
